# The Clinical Features of Patients with Chronic Hepatitis C Virus Infections Are Associated with Killer Cell Immunoglobulin-Like Receptor Genes and Their Expression on the Surface of Natural Killer Cells

**DOI:** 10.3389/fimmu.2017.01912

**Published:** 2018-01-05

**Authors:** Ariel Podhorzer, Melisa Dirchwolf, Andrés Machicote, Santiago Belen, Silvina Montal, Silvia Paz, Hugo Fainboim, Luis G. Podestá, Leonardo Fainboim

**Affiliations:** ^1^Instituto de Inmunología, Genética y Metabolismo (INIGEM-CONICET), Hospital de Clínicas José de San Martín, Universidad de Buenos Aires, Buenos Aires, Argentina; ^2^Hepatopatías Infecciosas, Hospital Francisco J. Muñiz, Buenos Aires, Argentina; ^3^Unidad de Cirugía Hepato-Biliar y Trasplante, Hospital Universitario Austral, Buenos Aires, Argentina; ^4^Departamento de Microbiología, Parasitología e Inmunología, Facultad de Medicina de la Universidad de Buenos Aires, Buenos Aires, Argentina

**Keywords:** chronic HCV, liver, killer cell immunoglobulin-like receptor, natural killer cells, T cells

## Abstract

Killer cell immunoglobulin-like receptor (KIR) genes are known to play a role in the acute phase of hepatitis C virus (HCV) infection. The present study investigated their roles in chronic HCV (CHCV) infection by analyzing the phenotypes and function of natural killer (NK) and T cells that express KIRs. T cells from CHCV patients showed a more differentiated phenotype, and NK cells exhibited an activated profile. These observations are consistent with the increased expression of the degranulation marker CD107a observed after PMA stimulation. We explored the correlations between the expression of KIR genes and lectin type-C receptors with clinical factors that predict progression to fibrosis and cirrhosis. The expression levels of KIR2DS3 and the functional alleles of KIR2DS4-FL were increased in patients with intermediate and high viral loads. Homozygous KIR2DS4 was also associated with the presence of cirrhosis. In the group of individuals with a shorter infection time who developed cirrhosis, we detected decreased expression of KIR3DL1 in CD56^dim^ NK cells in the presence of its ligand. Similarly, in the group of patients with late CHCV infections complicated with cirrhosis, we detected lower expression of the strong inhibitory receptor NKG2A in CD56^bright^ NK cells. We also detected an increase in NKG2C expression in CD56^dim^ NK cells in CHCV patients who displayed high necroinflammatory activity. Decreased KIR3DL2 expression in CD56^dim^ and CD56^bright^ NK cells was associated with a high body mass index, and KIR3DL2 expression may be one factor associated with the more rapid progression of CHCV to fibrosis in patients.

## Importance

Our results indicate that increased expression of an activated receptor or decreased expression of the inhibitory counterpart may be associated with a worse clinical evolution during the chronic phase of the hepatitis C virus (HCV) infection, in contrast to the acute phase of HCV infection. These changes should be evaluated in the context of a more differentiated/activated state of peripheral blood and liver T cells and natural killer (NK) cells and their increased capacity to degranulate, which may reflect a potential increase in cytotoxic activity.

## Introduction

Hepatitis C virus infects over 170 million people worldwide (1). During the acute phase, a small proportion of individuals can naturally clear HCV using NK cells. Based on studies of health-care workers exposed to small amounts of HCV, activated NK cells may participate in controlling acute infection and subsequent HCV-specific T-cell response (2). NK cells from patients who later cleared the infection have a greater antiviral effect *in vitro* than NK cells from patients who progressed to a chronic HCV (CHCV) infection (3). According to early genetic studies, spontaneous HCV clearance is observed in patients with the KIR2DL3/HLA-C1 compound genotype, which results in a lower activation threshold for NK cells (4). NK cells are traditionally regarded as first-line effectors of the innate immune response and may also have a distinct role in chronic infection. If early resolution does not occur, NK cell activity decreases and the adaptive immune system begins to respond in a specific way. However, if the adaptive immune system does not succeed in eradicating the virus, the infection becomes a persistent and chronic infection in the presence of continuous viral replication, potentially leading to the development of liver cirrhosis and hepatic cellular carcinoma. The innate immune response to an infection is likely to influence the type of adaptive immune response that develops and will ultimately determine whether the virus is cleared or develops into a chronic infection [reviewed in Rehermann (5)]. NK cells are known to kill HCV-infected hepatocytes and produce IFN-γ, the main antiviral cytokine (6). NK cells are divided into functionally distinct subsets based on their level of CD56 surface expression: the mainly cytotoxic CD56^dim^ population and the more immunoregulatory cytokine-producing CD56^bright^ NK cell subset. The functions of both NK cell subsets are modulated by inhibitory and activating signals provided by distinct classes of receptors. Inhibitory receptors include the polymorphic system, killer cell immunoglobulin-like receptors (KIR) (7), and a member of the C-type lectin-like receptor family, CD94/NKG2A, which recognizes HLA-E (8). Activating receptors include natural cytotoxicity-inducing receptors (NKp30, NKp44, and NKp46), the lectin-like receptors NKG2C (expressed as a dimer with CD94), and NKG2D, the signaling lymphocyte activation molecule family receptors (9), and the FcγRIIIa receptor (CD16), which mediates antibody-dependent cytotoxicity (10). The role of KIR genes in the chronic stage of infection has been mostly identified at the genomic level (11, 12) or has been associated with the role of HCV in the development of HCV-associated diseases (13, 14).

The role of T cells in HCV infection has been studied extensively (15). Because the liver is the target of HCV infection, studies aiming to understand the difference between liver and peripheral blood T cells are necessary. Liver CD3^+^ cells are characterized by a high percentage of CD3^+^CD8^+^ cells and a subset of CD3^+^CD56^+^ cells (16, 17). CD8^+^ T cells exist in at least three different states of reactivity: naïve CD8^+^ T cells with low reactivity, activated (effector) CD8^+^ T cells with high reactivity, and memory CD8^+^ T cells with intermediate reactivity. The overall memory CD8^+^ T cell compartment consists of central and memory subsets, which are recognized based on their phenotype and function (18, 19). Recently, we reported high levels of KIR receptor expression in peripheral blood and liver CD3^+^CD56^+^ cells and in liver T cells (20).

The aim of the present work was to examine the correlations between the KIR genotype and KIR protein expression with clinical aspects related to the development of CHCV infection. In particular, we investigated the roles of NK and T cells in CHCV patients and analyzed the KIR genotype and KIR protein expression on peripheral blood and liver cells. We next explored the putative associations of KIR genes and lectin-type receptors with clinical factors that predict progression to fibrosis and cirrhosis.

## Patients and Methods

The cohort included 273 healthy subjects and 351 adult individuals with CHCV infection, 117 of whom had not received any treatment. In addition, liver samples were collected from 23 healthy adult individuals and 6 CHCV subjects who were undergoing transplantation. Patients and controls were recruited from the Hepatology Services of Hospital de Enfermedades Infecciosas “F. J. Muñiz,” Hospital de Clínicas “José de San Martin,” and Hospital Universitario Austral. The healthy controls were matched with patients according to gender and ethnicity. All subjects belong to a homogeneous Latin American Caucasian population that is primarily composed of second- or third-generation Argentines, most of whom have a Spanish or Italian background. A small number of Amerindians and black individuals are living in Argentina but these individuals were not enrolled in the present study. All controls were examined and found to be negative for HIV, hepatitis B virus (HBV), and HCV. All CHCV patients were negative for HIV and HBV.

The study was approved by the Investigation and Ethics Committee and Institutional Review Board of the Hospital de Clínicas José de San Martín and the experiments were performed in accordance with the ethical guidelines of the 1975 Declaration of Helsinki. All samples obtained during the liver transplant were identified by a transplant procedure number provided by INCUCAI and did not include the name of the donor. None of the transplant donors were from a vulnerable population and all donors or next of kin provided voluntary written informed consent.

### Clinical Characteristics of Patients with CHCV Infections

A summary of the clinical characteristics is provided in Table 1. Serum anti-HCV antibody titers of all patients were determined using the third-generation ELISA technique (4.0 Murex-Abbot), and detectable levels of viral RNA were measured using qualitative PCR (AMPLICOR^®^ Hepatitis C Virus Test 2.0). Only peripheral blood samples from patients who had not been treated were used to analyze the expression of markers, functional capacity, and correlations with factors predictive of evolution to fibrosis. In contrast, samples from treated patients were included in the genomic study. Liver samples from individuals with CHCV infection were obtained during the surgical procedure for the liver transplant. Data regarding sex, body mass index (BMI), the time when patients became infected (when this information was available), viral load, liver transaminase levels, inflammatory activity, and the degree of fibrosis were obtained to examine factors that predicted clinical progression. For various reasons related to the origin of the samples, we were not able to include all these data from the whole cohort of CHCV patients. Viral load was measured in 96 patients using the qRT-PCR technique with the COBAS^®^ TaqMan^®^ HCV Test v2.0 kit (Roche). Individuals were separated into three groups according to the obtained viral load: 12 individuals with a low viral load (<100,000 IU/ml), 25 individuals with a moderate viral load (between 100,000 and 500,000 IU/ml) and 59 individuals with a high viral load (≥500,000 IU/ml).

**Table 1:** Clinical characteristics of the patients with chronic HCV (CHCV) infections.

CHCV clinical features	Median or factor (*n*)	Range
Years	51 years	(20–77)
Sex	F:166, M:185	
Genotype	1 (*n*: 37), 2 (*n*: 14), 3 (*n*: 16), 4 (*n*: 3)	
Body mass index	26 (*n*: 47)	(18–40)
Viral load	770,442 IU/ml (*n*: 96)	(500–2,000,000)
Transaminases	1.8 (ratio) (*n*: 291)	(0.6–8.4)
Time since infection	27 years	(1–61)
Fibrosis	METAVIR: F0 (*n*:7), F1 (*n*:18), F2 (*n*:13), F3 (*n*:8), F4 (*n*:9)	
	FibroScan: F0 (*n*:16), F1 (*n*:13), F2 (*n*:20), F3 (*n*:8), F4 (*n*:20)	
	Ishak: E1 (*n*:29), E2 (*n*:3), E3 (*n*:14), E4 (*n*:3), E5 (*n*:4), E6 (*n*:32)	
Necroinflammatory activity	METAVIR: A0 (*n*:2), A1 (*n*: 27), A2 (*n*:17), >A3 (*n*:9)	
Cirrhosis	171 individuals	

The COBAS^®^ HCV GT kit (Roche) was used to analyze the different viral genotypes; this kit distinguishes between genotypes 1 and 6 using a highly sensitive real-time PCR assay. The analysis of 115 CHCV patients showed the following distribution of viral genotypes: genotype 1:37 cases, genotype 2:14 cases, genotype 3:16 cases, and genotype 4:3 cases. BMI was obtained from 47 patients, 22 of whom had normal weight (BMI ≤ 25) and 25 were overweight (BMI > 25). The transaminase activities of SGOT or aspartate aminotransferase (AST) and SGPT or alanine aminotransferase (ALT) were measured in 291 HCV patients, 90 of whom had normal or slightly increased activity levels and 201 had values elevated by more than twofold relative to the upper limit of normal.

To evaluate the degree of inflammation and fibrosis, 140 liver biopsies were performed using a percutaneous or trans-jugular method. Fifty-five cases were evaluated using the METAVIR system. This system classifies fibrosis in stages ranging from F0 to F4, and in the present study, higher stages (F3 and F4) were considered to indicate cirrhosis. The METAVIR system also classifies necroinflammatory activity into stages A0–A3; in our study, stages A2 and A3 were considered to indicate high necroinflammatory activity and were associated with a more rapid progression to cirrhosis (21). Eighty-five individuals were evaluated using the Ishak score, which classifies fibrosis in stages ranging between E1 and E6. For this study, we considered the stages E5 and E6 to indicate cirrhosis. In 77 individuals, the degree of fibrosis was obtained using the non-invasive method of transient elastography (Fibroscan^®^), which classifies fibrosis in stages ranging from F0 to F4, and stages F3 and F4 were considered to indicate cirrhosis. Finally, in 134 patients, the presence or absence of cirrhosis was simply determined by the combination of the results of clinical studies and ultrasonography.

### Collection of Perfused Liver Samples

Samples were collected from donor livers and recipients using methods previously described by Kelly et al. (22). During orthotopic liver transplantation, 23 samples from donor livers (Table 2) and 6 samples from CHCV-infected recipients were collected at the Austral University Hospital. All samples were identified by a transplant procedure number provided by INCUCAI that did not include the name of the donor and were collected from the Austral University Hospital, Buenos Aires, Argentina. After brain death, the procuration team working in the intensive care unit required the next of kin to sign the donor consent form for transplantation and participation in the research project.

**Table 2:** Donor data: age, sex, and cause of death.

Age	Sex	Cause of death
60	F	Hemorrhagic stroke
56	F	Hemorrhagic stroke
25	M	Traumatic brain injury
40	F	Hemorrhagic stroke
50	F	Hemorrhagic stroke
28	M	Traumatic brain injury
43	F	Traumatic brain injury
20	M	Traumatic brain injury
58	F	Hemorrhagic stroke
20	M	Hypoxic brain injury
19	M	Traumatic brain injury
19	M	Traumatic brain injury
35	M	Hemorrhagic stroke
49	M	Hemorrhagic stroke
55	F	Hemorrhagic stroke
33	M	Traumatic brain injury
27	M	Traumatic brain injury
18	M	Traumatic brain injury
23	M	Traumatic brain injury
52	M	Hemorrhagic stroke
56	M	Hemorrhagic stroke
45	F	Hemorrhagic stroke
55	F	Hemorrhagic stroke

During retrieval (at the time of exsanguination), the donor aorta and superior mesenteric vein were flushed with University of Wisconsin (UW) solution (Bristol-Myers Squibb, Uxbridge, UK) or with HTK (histidine–tryptophan–ketoglutarate) solution. After excising the organ, the liver was again flushed with UW solution until all blood was removed and the perfused solution appeared clear. At implantation and after completing the upper inferior cava anastomosis, livers were flushed with Ringer’s lactate solution through the portal vein at 4°C to wash out the UW before reperfusion. The perfused hepatic fluid was collected from the inferior cava vein (600–1,200 ml).

### Liver Samples from Patients with CHCV Infections

After each operation, the liver from the CHCV patient was placed in a container with a physiological solution, and 500 ml of lactated Ringer’s solution was infused through the portal vein; after traversing the organ, this solution was recovered through the suprahepatic veins. The liver remained in this solution during the transplant process. Subsequently, this solution was discarded, and the infusion of 500 ml of lactated Ringer’s solution through the portal vein was repeated; the solution from the second infusion was then collected.

### Mononuclear Cell Isolation

Peripheral blood mononuclear cells (PBMCs) from adult healthy controls and liver mononuclear cells (LMCs) from healthy cadaveric donors and recipients were obtained using Ficoll-Hypaque density gradient centrifugation (GE Healthcare Biosciences, Uppsala, Sweden).

### Monoclonal Antibodies and Flow Cytometry

Peripheral blood mononuclear cells and LMCs were stained with unconjugated antibodies against KIR 3DL1/3DL2 (clone 5.133, courtesy of Dr. M. Colonna), KIR 2DL2/2DS2/2DL3 (clone CHL, courtesy of Dr. S. Ferrini), KIR 2DL1/2DS1 (clone HPMA4), KIR 2DL1/2DS1/2DS3 (clone HP3E4), CD94 (clone 3D9), and NKG2A (clone Z199, courtesy of Dr. M. Bottet) or unconjugated IgG1, IgG2a, IgG2b, and IgM isotype control antibodies (Becton Dickinson, San Jose, CA, USA). In addition, mouse monoclonal antibodies against 3DL1-FITC, 2DL3-PE (Becton Dickinson, San Jose, CA, USA), 2DS4-PE, NKG2C-PE (R&D Systems, MN, USA), NKp44-PE, NKp46-PE, 2B4-FITC, CD16-FITC, CD57-APC, CD8-PE/APC, CD4-FITC, CD45RA-PE-Cy7, CD28-PE, CD27-PE-CF594, CCR7-FITC, CD11b-FITC, CD161-APC/PE, HLA-DR-FITC, and labeled IgG1 and IgG2a isotype control antibodies (Biolegend, San Diego, CA, USA) were used. Cells were incubated with the above mentioned antibodies at room temperature (RT) for 30 min, then were washed twice with 1 ml of 1× PBS and centrifuged at 500 *g* for 5 min. For unconjugated antibodies, a subsequent incubation with 3 µl of FITC- or PE-conjugated antimouse immunoglobulin antibody (Dako, Glostrup, Denmark) was performed for 30 min at RT. After washing, 3 µl of normal mouse serum was added. Cells were incubated with 3 µl of anti-CD3 (Becton Dickinson, San Jose, CA, USA) conjugated to PerCP/FITC and 5 µl of anti-CD56 conjugated to PE/APC (Becton Dickinson, San Jose, CA, USA) or to BV421 (Biolegend, San Diego, CA, USA) for 15 min at RT to analyze labeling corresponding to the NK, NKT (CD3^+^CD56^+^), and T cells. A suitable fluorochrome combination was performed for each labeling scheme. Liver samples were also incubated with the anti-CD45 pan-leukocyte marker (Becton Dickinson) conjugated to APC-H7. After washes with 1× PBS, cells were fixed with 2% paraformaldehyde and subsequently analyzed using a FACSAria II flow cytometer (Becton Dickinson). The results were analyzed using FlowJo 7.6.2 software (Tree Star, Inc., Ashland, OR, USA). All analyses of a particular population of interest were based on the gating of at least 100,000 events. The surface expression of three KIR receptors was indirectly inferred as follows: the antibody 3DL1/3DL2 showed two populations with different mean fluorescence intensities, the lower population corresponded to 3DL2 and the upper population to 3DL1. In all cases, the expression of the upper population was compared with the monoclonal antibody against 3DL1 and a significant difference was not detected. The expression of 2DS3 was identified by subtracting the expression detected using the 2DL1/2DS1/2DS3 antibodies from 2DL1/2DS1 expression. The expression of 2DS1 was deduced by subtracting the expression of 2DL1 from the expression detected using the 2DL1/2DS1 antibody. The expression of 2DL2/2DS2 was inferred after subtracting the expression detected by the anti-2DL3 antibody to the expression detected by the anti-2DL2/2DS2/2DL3 antibody. In all cases, the presence of the KIR gene was verified. When using polyclonal antibodies, the correct method to deduce the expression of a single KIR protein is to use polyclonal and monoclonal antibodies together in the same tube (23); however, because of the limited number of fluorochrome combinations, we performed the reactions in different tubes and inferred the expression of single KIR proteins (see Figure S1 in Supplementary Material).

We distinguished between apoptotic and viable cells based on differences in forward and side scatter, and the results showed a good correspondence with the results obtained using FITC-annexin staining (24, 25). We previously assessed the quality of the results through dead cell staining, showing that dead cell discrimination by forward/side scatter displayed an excellent correlation with the results obtained using the LIVE/DEAD^®^ Fixable Aqua Dead Cell Stain Kit, which specifically stains lymphocytes.

### KIR and HLA-A, B, and C Typing by PCR Sequence-Specific Oligonucleotide Probing (SSOP)

The conditions used to identify the presence or absence of each KIR gene have been documented previously (20). Briefly, two PCR amplifications were performed: PCR-1 amplified the combined D1 and D2 domains and PCR-2 amplified the transmembrane and cytoplasmic regions. Nineteen 5′-digoxigenin-labeled probes were used in the SSOP approach, with 13 for PCR-1 and 6 for PCR-2. The KIR gene content in each individual was inferred after analyzing the combinations of all probes. The KIR2DS4 gene was amplified by PCR with previously described primers to analyze the cell membrane-anchored receptor (designated KIR2DS4-FL or FL) and a truncated soluble protein (designated KIR1D), which is generated when exon 5 contains a 22-bp deletion. HLA-A, B, and C genotyping was performed for sequences from exons 2 and 3. The primers and conditions for PCR amplification were the same as those described by Cereb et al. (26). HLA-C SSOP typing was also conducted using two probes (5′-digoxigenin label); one of the probes (5′-TGACCGAGTGAACCTGC-3′) was specific for the HLA-C alleles that belong to the C1 group (Asn 80), and the other probe (sequence: 5′-ACCGAGTGAGCCTGCG-3′) anneals with the HLA-C alleles that belong to the C2 group (Lys 80).

### Functional Studies

Mononuclear cells obtained from the liver or peripheral blood were placed in 24-well plates at a density of 1 × 10^6^ lymphocytes per well and cultured in 1 ml of complete RPMI medium. Three wells were used for each sample according to the following scheme: (1) cells stimulated with the target antibody to study functionality, (2) cells stimulated with the isotype control, and (3) unstimulated cells that served as a negative control. Twenty microliters of the anti-CD107a antibody (Becton Dickinson) conjugated to FITC were added to wells 1 and 3 and 20 µl of the FITC-conjugated IgG1 (Becton Dickinson) isotype control was placed in well 2 as a control. PMA (phorbol 12-myristate 13-acetate; Sigma) was added to wells 1 and 2 at a final concentration of 25 ng/ml, and ionomycin (calcium salt from *Streptomyces conglobatus*; Sigma) was added at a final concentration of 0.5 µg/ml. The culture plate was incubated for 1 h at 37°C in a 5% CO_2_ atmosphere. One microliter of monensin (BD-Golgi Stop Protein Transport Inhibitor, BD Biosciences) was then added to all wells, and the cells were incubated for an additional 5 h. The contents of each well were transferred to FACS tubes; cells were washed with 1 ml of 1× PBS/A and centrifuged at 500 *g* for 5 min. In all samples, cell surface labeling was performed with the following monoclonal antibodies: 3 µl of anti-CD3 conjugated to PerCP (Becton Dickinson) and 5 µl of anti-CD56 conjugated to APC (Becton Dickinson). Pan-leukocyte anti-CD45 conjugated to APC-H7 (Becton Dickinson) was added to the liver samples. After a 20-min incubation at RT, cells were washed twice with 1× PBS/A. During intracytoplasmic labeling, cells were treated with 100 µl of 1× fixation and permeation solution (Cytofix/Cytoperm, BD Biosciences) for 20 min at 4°C, washed twice with 1 ml of 1× Perm/Wash buffer (Becton Dickinson), and centrifuged at 500 *g* for 5 min. Twenty microliters of IgG1 isotype PE control (Becton Dickinson) were added to well 2, and 20 µl of anti-IFNγ conjugated to PE (Becton Dickinson) were placed in wells 1 and 3; then, the samples were incubated for 30 min at 4°C. Cells were washed with 1× Perm/Wash buffer, the pellet was suspended in 200 µl of 1× PBS/A, and the cells were fixed with 2% paraformaldehyde. Finally, the cells were analyzed using a FACSAria II flow cytometer (Becton Dickinson).

### Statistical Analysis

The HLA allele frequencies and expression of KIR genes in the patients and controls were compared using Fisher’s exact test to evaluate significant differences, and the *p*-value was corrected with the Bonferroni method when appropriate. Additionally, 3 × 2 contingency tables were analyzed using the Chi-square test for independent samples or the maximum likelihood method when appropriate (viral load factor). The Mann–Whitney *U* test was used to compare independent groups. Spearman’s test was used to establish correlations. For all statistical analyses, we used GraphPad Prism 5 (GraphPad Software). Data are presented as medians and ranges. Reported *p*-values are two-tailed, and *p* < 0.05 was considered significant.

## Results

### Typing of HLA and KIR Genes in CHCV Patients

No significant differences in the typing of HLA-A, HLA-B, and HLA-C genes were observed between HCV patients (*n* = 351) and healthy individuals (*n* = 273) (Table 3).

**Table 3:** Genetic frequency expressed as percentage of HLA-A, HLA-B, and HLA-C, genes in healthy individuals and chronic HCV (CHCV) patients.

HLA-A	1	2	3	11	23	24	25	26	29	30	31	32	33	34	36	43	66	68	69	74	80															
Healthy Ind.	10	25	8	6	2	11	1	4	6	6	4	3	2	0	0	0	1	7	0	1	0															
CHCV	10	21	0	7	3	11	1	4	6	6	7	3	3	1	0	0	1	6	1	0	1															
HLA-B	7	8	13	14	15	18	27	35	37	38	39	40	41	42	44	45	46	47	48	49	50	51	52	53	54	55	56	57	58	59	67	73	78	81	82	83
Healthy Ind.	5	6	2	4	7	5	2	13	1	4	6	5	2	1	11	1	0	1	1	2	2	6	2	1	0	2	0	4	2	0	0	0	1	0	0	0
CHCV	6	4	2	6	6	5	2	13	1	3	5	5	1	1	12	2	0	1	1	3	2	7	3	1	0	1	1	2	2	0	0	0	0	0	0	0
HLA-C	1	2	3	4	5	6	7	8	12	13	14	15	16	17	18																					
Healthy Ind.	3	4	11	15	5	9	23	5	9	0	1	4	8	2	1																					
CHCV	4	4	10	15	5	8	22	6	9	0	1	4	9	2	1																					

*Fisher’s exact test*.

The KIR genotyping results did not show any differences from the data reported in a previous study (11) (Table 4). However, the typing of different KIR2DS4 alleles (27) revealed an increased frequency of the functional KIR2DS4 alleles (KIR2DS4-FL) in patients with CHCV infections (0.52 vs. 0.42 in healthy controls, *p* = 0.02) (Table 5). Similarly, the deleted forms (KIR1D) showed a decreased frequency of the homozygous KIR1D genotype and a clear tendency toward an increase in the frequency of the homozygous KIR 2DS4FL genotype in patients with CHCV infections.

**Table 4:** Genotype frequencies of killer cell immunoglobulin-like receptor (KIR) genes.

KIR	*n*	2DL1	2DL2	2DL3	2DL4	2DL5	3DL1	3DL2	3DL3	2DS1	2DS2	2DS3	2DS4	2DS5	3DS1	2DP1	3DP1
Healthy Ind.	273	0.95	0.61	0.85	1.00	0.55	0.95	1.00	1.00	0.40	0.58	0.29	0.95	0.35	0.40	0.95	1.00
Chronic HCV	351	0.97	0.60	0.88	1.00	0.58	0.93	1.00	1.00	0.45	0.59	0.34	0.90	0.38	0.47	0.97	1.00

**Table 5:** Genotype frequencies of the KIR2DS4-FL^+^ gene in chronic HCV (CHCV) patients and healthy individuals.

	*n*	2DS4 FL^**+**^	*2ds4 fl*^**+**^ homozygote	*2ds4 fl*^**+**^*/kir1d*	*kir1d* homozygote
Healthy Ind.	259	0.42	0.18	0.24	0.58
CHCV	316	0.52*	0.25	0.27	0.48*

*p < 0.05; Fisher’s exact test.

Killer cell immunoglobulin-likereceptors must recognize their ligands to perform their functions. Among the inhibitory KIRs, KIR2DL1 recognizes HLA-C2 allotypes containing a lysine at position 80 of the HLA-Cα_1_ domain and KIR2DL2/3 recognize HLA-C1 alleles containing Ser77/Asp80 (28, 29). However, KIR2DL2 and, to some extent, KIR2DL3 interact with some C2 alleles with weaker affinity than KIR2DL1. The genotype frequency of individuals with a known ligand is shown in Table 6. After analyzing the whole population of CHCV patients, the comparison between healthy individuals and CHCV patients showed no significant differences.

**Table 6:** Frequencies of killer cell immunoglobulin-like receptor (KIR) genes and their ligands.

	*2dl1^**+**^* HLA-C2	*2dl2^**+**^* HLA-C1	*2dl3^**+**^* HLA-C1	*3dl1^**+**^* HLA-Bw4	*3dl2^**+**^* HLA-A *03/*011	*2ds1^**+**^* HLA-C2	*2ds2^**+**^* HLA-C1	*2ds4 fl^**+**^* Putative ligands
Healthy Ind.	0.64	0.86	0.82	0.66	0.26	0.64	0.84	0.60
Chronic HCV	0.64	0.87	0.85	0.64	0.27	0.68	0.87	0.62

*The analyses were performed on CHCV patients and healthy controls possessing the KIR gene. The n value for each KIR gene was deduced from the genotype frequencies shown in Tables 4 and 5. Fisher’s exact test*.

### Expression of KIR Receptors in CHCV Patients

**Figure 1:**
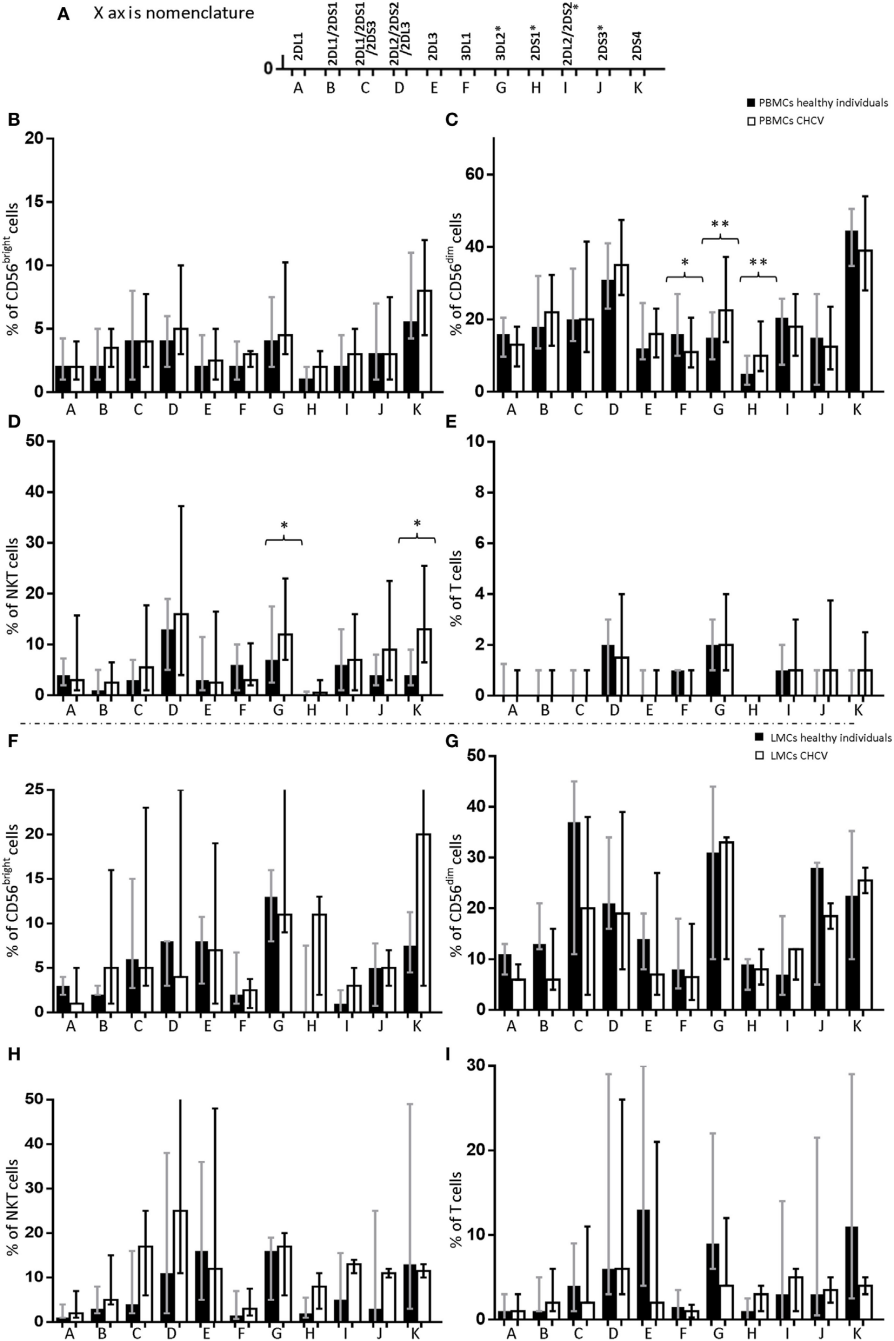
Differences in the expression of killer cell immunoglobulin-like receptor (KIR) genes in the following cell subsets from chronic HCV (CHCV) patients and healthy controls: **(A)** Each KIR on the X axis is represented by its corresponding letter. peripheral blood mononuclear cell (PBMCs): **(B)** CD56^bright^, **(C)** CD56^dim^, **(D)** NKT (CD3^+^CD56^+^), and **(E)** T cells; liver mononuclear cells (LMCs): **(F)** CD56^bright^, **(G)** CD56^dim^, **(H)**, NKT (CD3^+^CD56^+^), and **(I)** T cells. Each bar shows the median expression of positive cells for each KIR and the IQR. *p < 0.05, **p < 0.01, Mann–Whitney *U* test.

In contrast to the genotype frequency, the expression of KIR genes in PBMCs from patients with CHCV infections showed differences compared with PBMCs from healthy controls. NK (CD56^+^CD3^−^), NKT (CD3^+^CD56^+^), and T (CD3^+^) cells were analyzed. CD56^dim^ cells from CHCV patients carrying the KIR gene showed higher expression of KIR2DS1 (10.1 vs. 5.3% in CD56^dim^ cells from healthy controls, *p* = 0.003) (Figure 1C) and KIR3DL2 (22.5 vs. 15.1% in healthy controls, *p* = 0.009) (Figure 1C) and lower expression of KIR3DL1 (11.4 vs. 16.4% in healthy controls, *p* = 0.04) (Figure 1C). CD3^+^CD56^+^ cells also showed increased expression of KIR3DL2 (12.3 vs. 7.1% in healthy controls, *p* = 0.02) and KIR2DS4 (13.3 vs. 4.0% in healthy controls, *p* = 0.01) (Figure 1D). No differences were found in the expression of KIRs within CD56^bright^ NK cells (Figure 1B) and T cells (Figure 1E). Importantly, PBMCs collected from CHCV patients, and healthy LMCs were collected from untreated individuals. LMCs from CHCV patients were collected before liver transplantation on previously treated patients. However, LMCs from CHCV patients showed levels of KIR expression that were similar to those in LMCs from healthy controls (Figures 1F–I). Expression was measured only in individuals carrying the KIR gene, which partially explains the small number of CHCV-infected liver samples examined.

### NK and T Cell Populations in Patients with CHCV Infections

**Figure 2:**
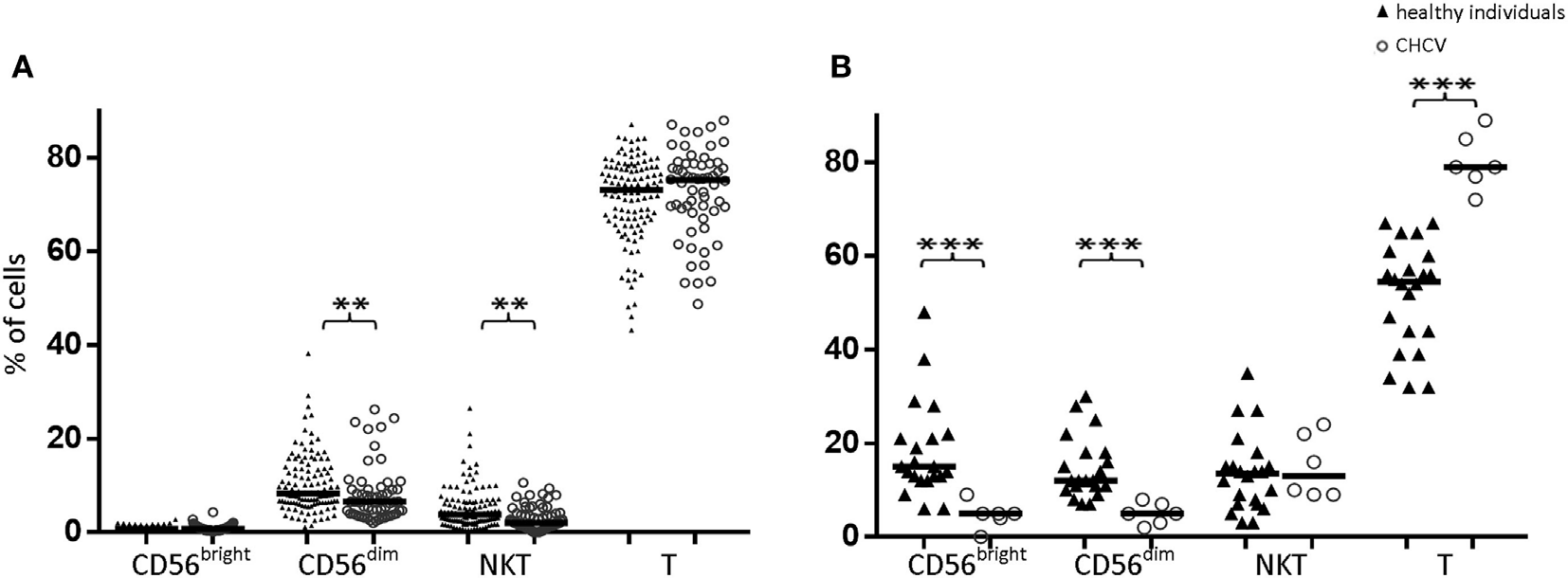
**(A)** Comparison of CD56^bright^, CD56^dim^, NKT (CD3^+^CD56^+^), and T lymphocyte subpopulations in peripheral blood between healthy individuals and chronic HCV (CHCV) patients. **(B)** Comparison of liver cell subpopulations between healthy individuals and CHCV patients. The results are expressed as percentages of positive cells within each subpopulation. Black lines indicate median expression. **p < 0.01, ***p < 0.001, Mann–Whitney *U* test.

Compared with those from healthy controls, PBMCs from CHCV patients showed decreased frequencies of CD56^dim^ NK cells (6.5 vs. 8.3%, *p* = 0.002) and CD3^+^CD56^+^ cells (2.0 vs. 3.8%, *p* = 0.001) (Figure 2A). Similarly, decreased frequencies of CD56^dim^ NK cells (5.0 vs. 12.2% in CD56^dim^ cells from healthy controls, *p* < 0.001) and CD56^bright^ cells (5.4 vs. 15.0% in healthy controls, *p* < 0.001) (Figure 2B) were detected in the liver of CHCV patients, but the CD56^dim^/CD56^bright^ ratio was maintained (50% of each subset). Livers of CHCV patients showed significant infiltration of T lymphocytes, suggesting that the decrease in the frequency of NK cells occurred at the expense of an increase in the frequency of T lymphocytes (79.1 vs. 54.5% in peripheral blood, *p* < 0.001). The lack of changes in the frequency of liver CD3^+^CD56^+^ cells indicates that the increase in the frequency of T cells observed in CHCV patients involved T cell subsets aside from CD3^+^CD56^+^ cells (Figure 2B).

### Expression of Other NK Cell Markers in CHCV Patients

**Figure 3:**
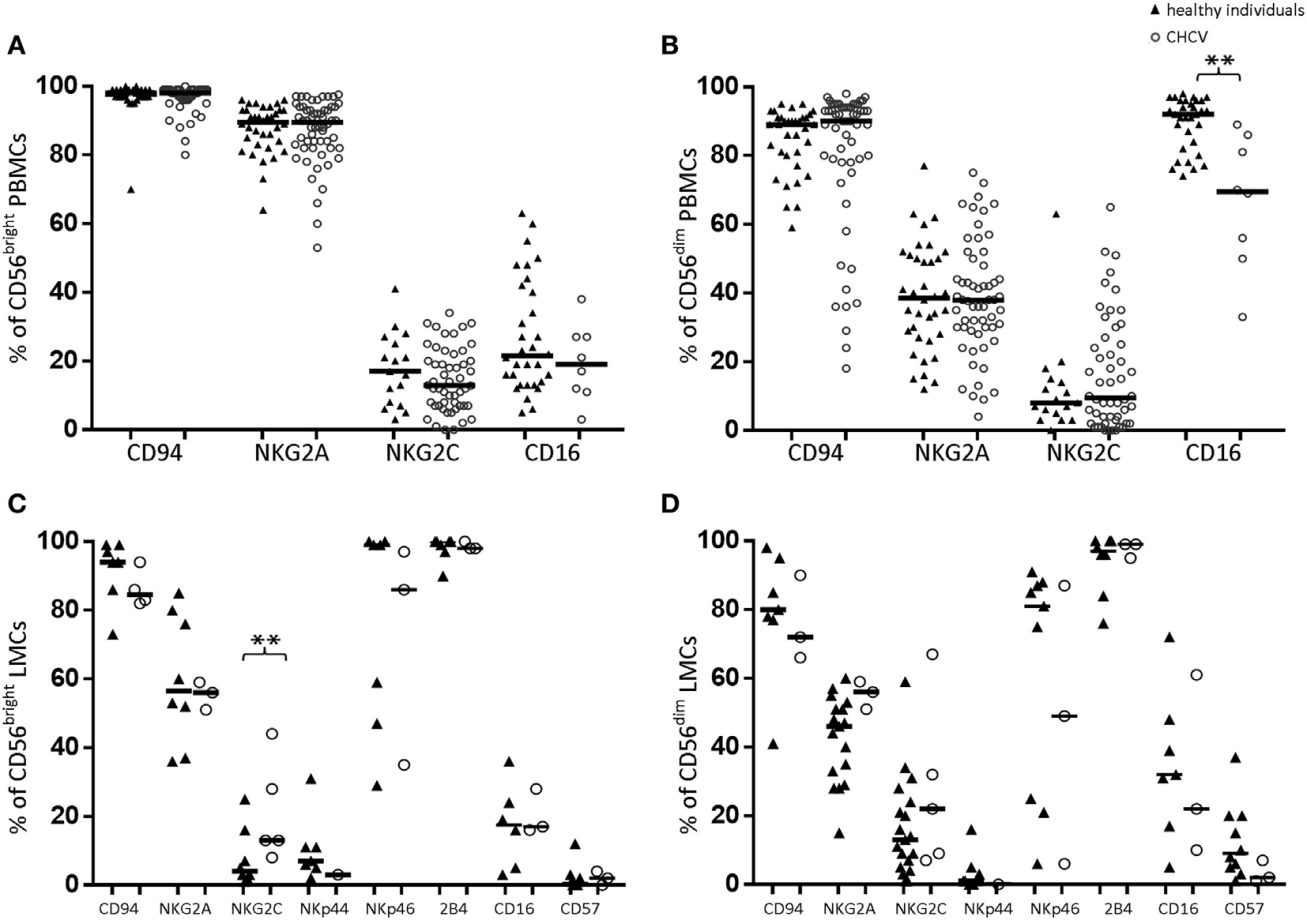
Expression of natural killer (NK) cell markers in peripheral blood lymphocytes **(A,B)** and liver mononuclear cells (LMCs) **(C,D)** from healthy and chronic HCV (CHCV)-infected individuals. The black line indicates median expression of the specific marker within the CD56^bright^ and CD56^dim^ populations. **p < 0.01, Mann–Whitney *U* test.

Compared with those from healthy controls, PBMCs-CD56^bright^ NK cells from CHCV patients showed no differences in the expression of CD94, NKG2A, NKG2C or CD16 (Figure 3A). In contrast, PBMCs-CD56^dim^ NK cells from CHCV patients showed a decreased frequency of CD16 expression (69.5 vs. 92.0%, *p* = 0.002; Figure 3B). Similarly, liver CD56^dim^ cells maintained the same low expression of CD16 in liver healthy controls (Figure 3D), which implies a potentially lower capacity to exert ADCC. We detected more frequent expression of the activating receptor NKG2C in the liver CD56^bright^ cells from patients with CHCV infections (13.2 vs. 4.2% in healthy controls, *p* = 0.04; Figure 3C).

### NK Receptors and the Clinical Course of HCV Infection

We next evaluated the correlations of altered expression of NK receptors with clinical features of CHCV patients.

#### Association with BMI

**Figure 4:**
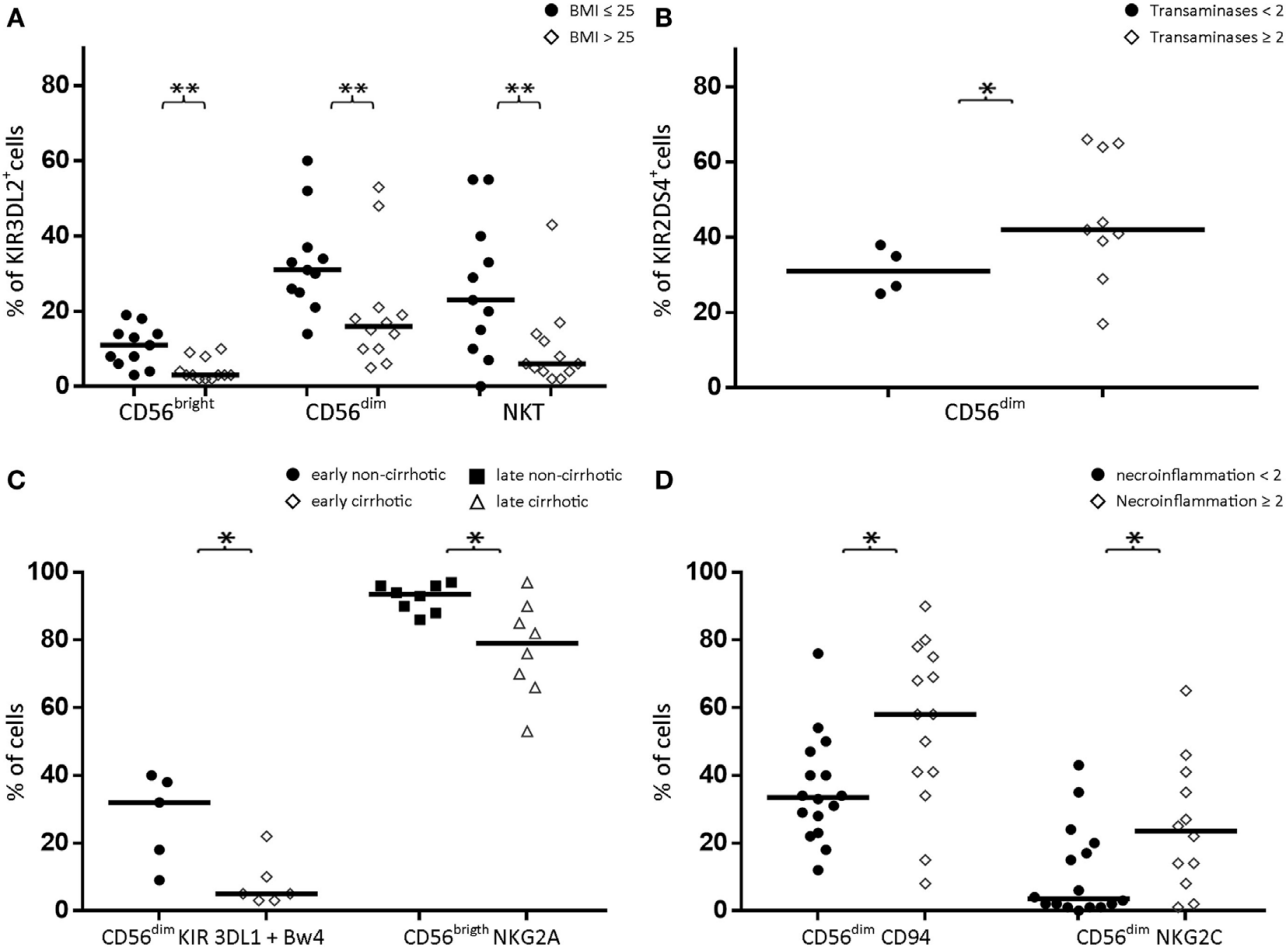
**(A)** Expression of KIR3DL2 in CD56^bright^, CD56^dim^, and NKT (CD3^+^CD56^+^) cell populations in peripheral blood from patients with normal body mass index (BMI) (≤25, *n* = 11) or elevated BMI (>25, *n* = 12). **(B)** Expression of KIR2DS4 in peripheral blood CD56^dim^ cells from patients with normal (<2) or elevated (≥2) liver transaminase levels. **(C)** Decreased expression of KIR3DL1 + Bw4 in CD56^dim^ cells from early cirrhotic patients (left) and decreased expression of NKG2A in CD56^bright^ cells from late-infected patients. **(D)** Increased expression of CD94 and NKG2C in peripheral blood CD56^dim^ cells from chronic HCV patients with high levels of necroinflammatory activity (≥2) compared with patients with low necroinflammatory activity (<2). Black lines indicate the medians of positive cells. *p < 0.05, **p < 0.01, Mann–Whitney *U* test.

KIR3DL2 expression was decreased in CD56^bright^ NK cells, CD56^dim^ NK cells, and CD3^+^CD56^+^ cells from CHCV patients with high BMI (>25) (3 vs. 11% in those with normal BMI, *p* = 0.002; 16 vs. 31% in those with normal BMI, *p* = 0.009; and 6 vs. 23% in those with normal BMI, *p* < 0.02, respectively; Figure 4A). KIR3DL2 expression was further increased in CHCV patients with normal BMI compared with healthy subjects. Furthermore, an association was observed between BMI and KIR3DL2 expression in CD56^bright^ NK cells and CD56^dim^ NK cells when the variables were expressed as continuous values (*r* value = 0.63, *p* = 0.001, and *r* value = 0.45, *p* = 0.03, respectively; Spearman’s test).

#### Association with Transaminase (SGPT/SGOT) Activity Levels

Consistent with the increased frequency of KIR2DS4-FL gene expression in CHCV patients, its expression was detected in peripheral blood CD56^dim^ NK cells with elevated transaminase levels (more than twofold higher than the upper limit of normal, Figure 4B) (42 vs. 31% in cells with normal transaminase levels, *p* < 0.05). However, the *r* value calculated using Spearman’s test was not significant when the variables were expressed as continuous values, although a trend was observed (*r* value 0.40, *p* = 0.17, Spearman’s test).

#### Association with the Time of Infection

Among CHCV patients, we were able to identify the approximate time of the infection in a subgroup (most cases were due to intravenous drug addiction or transfusion of blood). In our cohort, the median time that had elapsed since infection was 27 years. According to the time since HCV infection, patients were divided into early-infected (less than 27 years) and late-infected subgroups (more than 27 years).

As described above, inhibitory KIR3DL1 expression was decreased in the whole CHCV-infected patient cohort. Furthermore, KIR3DL1 expression was also decreased in peripheral blood CD56^dim^ NK cells (together with its Bw4 ligand) from the group of early-infected patients who developed cirrhosis (5 vs. 32% in patients without cirrhosis, *p* = 0.03; Figure 4C). Within the group of late-infected patients, individuals with cirrhosis displayed lower expression of the strong inhibitory receptor NKG2A in peripheral blood CD56^bright^ NK cells (79 vs. 94% in patients without cirrhosis, *p* = 0.02, Figure 4C). However, KIR3DL1 and NKG2A did not show correlations with the time of infection when the variables were expressed as continuous variables (Spearman’s test). Thus, we postulated that lower KIR3DL1 expression during early infection or NKG2A expression during late infection may contribute to progression to cirrhosis.

#### Association with Necroinflammatory Activity

Chronic HCV patients with high necroinflammatory activity (a METAVIR score ≥ 2) are expected to rapidly progress to fibrosis. Patients presenting high necroinflammatory activity displayed an increase in the expression of the CD94/NKG2C activator heterodimer in their peripheral blood CD56^dim^ NK cells (58 vs. 34% in those with lower necroinflammatory activity, *p* = 0.01 for CD94, and 24 vs. 3.5%, *p* = 0.04 for NKG2C; Figure 4D). Moreover, the *r* value calculated using Spearman’s test was not significant when the variables were expressed as continuous values.

#### Association of the Expression of the KIR2DS3 and KIR2DS4-FL Genes with the Viral Load and Cirrhosis

**Figure 5:**
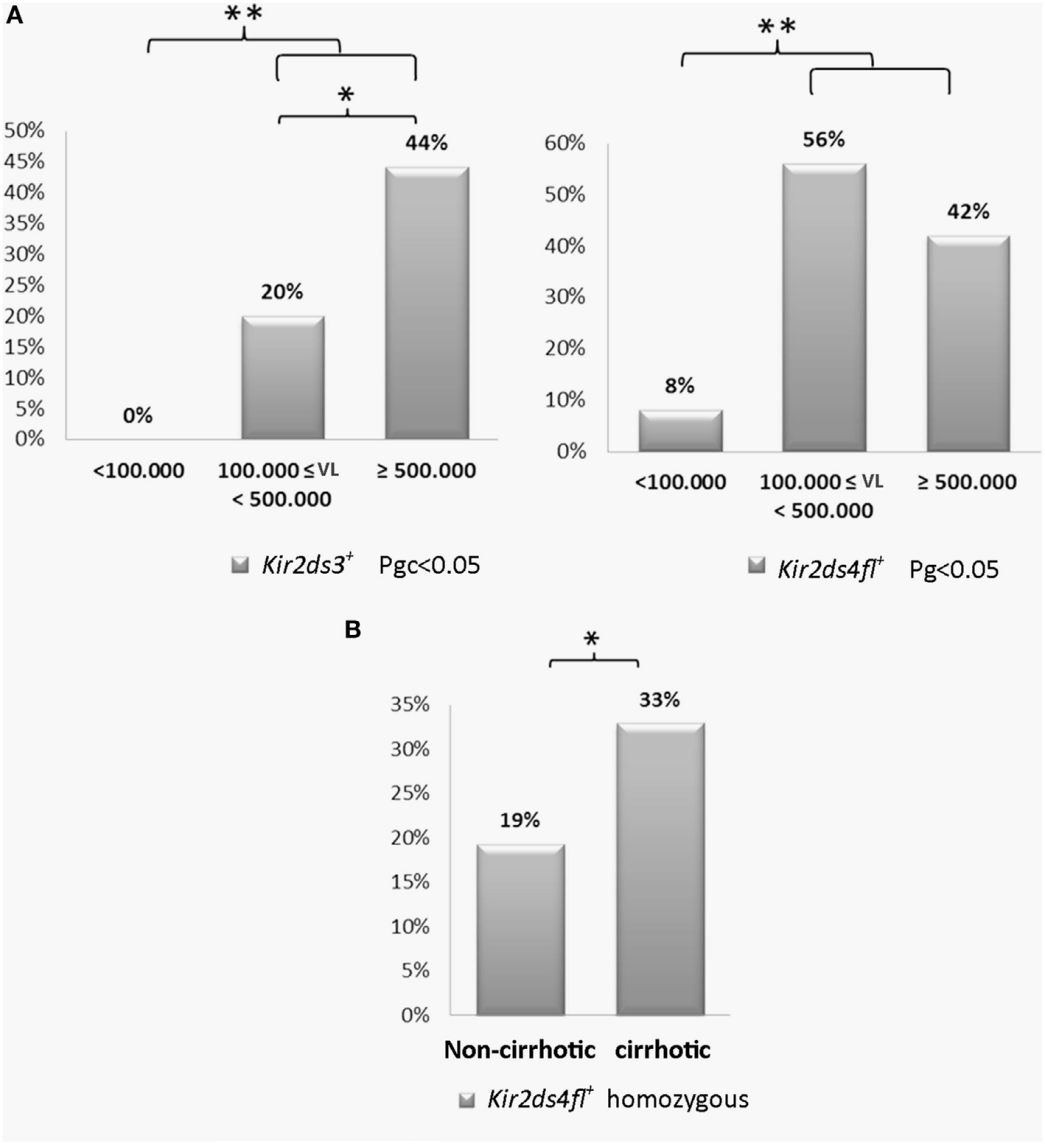
**(A)** Frequency of the KIR2DS3 and killer cell immunoglobulin-like receptor (KIR) 2DS4-FL genotypes in patients with low (<100,000 IU/ml, *n* = 12), intermediate (100,000–500,000 IU/ml, *n* = 25), and high (≥500,000 IU/ml) viral loads (*n* = 59). **(B)** Frequency of the homozygous KIR2DS4-FL genotype in cirrhotic (*n* = 88) and non-cirrhotic patients (*n* = 119). The frequencies were multiplied to be expressed as a percentage. *p < 0.05, **p < 0.01, ***p < 0.001; Chi-square test analyzed using the maximum likelihood method for low viral load vs. intermediate and high viral loads and for intermediate viral load vs. high viral load (Pgc < 0.05). For the analysis in **(A)**, the *p*-value was corrected using Bonferroni’s method. Fisher’s exact test was used for the analysis shown in **(B)**.

The viral load was arbitrarily grouped into three levels: low < 100,000 IU/ml, intermediate between 100,000–500,000 IU/ml and high ≥500,000 IU/ml. CHCV patients with a high viral load had a higher frequency of the KIR2DS3 genotype compared with patients with an intermediate viral load (44 vs. 20%; *p* < 0.05, OR: 3.2; Figure 5A). Strikingly, KIR2DS3, which is present in 29% of the Argentinian population (30), was not detected in any patients with a low viral load. Furthermore, the differences in the frequency of the KIR2DS3 genotype in patients with low viral loads and patients with intermediate and high viral loads were even more significant (44 and 20 vs. 0%, *p* = 0.04, OR: 9.8). Similarly, an increased frequency of the KIR2DS4-FL genotype was observed in patients with intermediate and high viral loads compared to patients with low viral loads (56 and 42 vs. 8%, *p* < 0.01, OR: 9.8; Figure 5A). Based on these findings, the presence of the activating genes 2DS3 and 2DS4-FL was associated with a high viral load. Furthermore, homozygosity for the KIR2DS4-FL genotype was also associated with progression to cirrhosis (33 vs. 19% in the non-cirrhotic group, *p* = 0.03, OR: 2.1; Figure 5B).

##### Sex

We did not detect differences according to sex (185 men and 166 women).

### Functional Studies of NK and T Cells in the Livers of Patients with CHCV Infections

**Figure 6:**
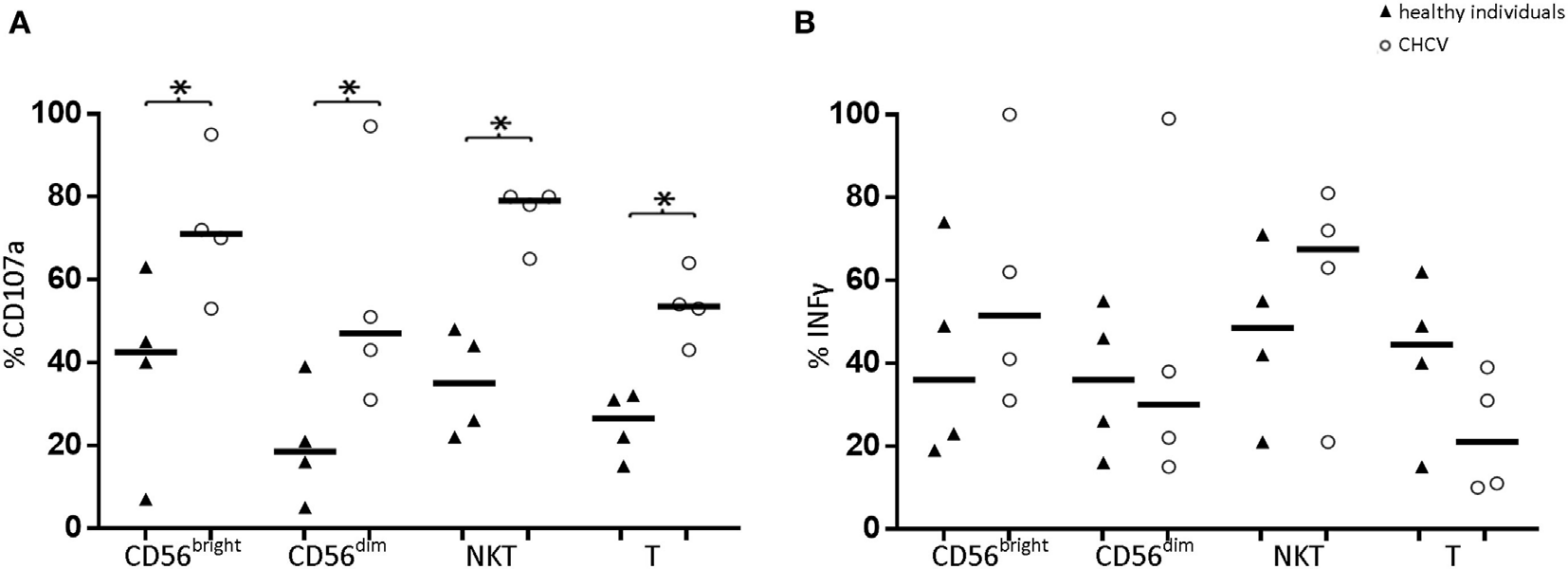
**(A)** Comparison of the cytotoxic capacity (indirectly measured as CD107a expression) and **(B)** secretory capacity (evaluated by measuring IFNγ production) of liver lymphocytes between healthy individuals (*n* = 4) and chronic HCV (CHCV) patients (*n* = 4). Black lines indicate the medians of cells positive for the different populations CD56^bright^, CD56^dim^, NKT (CD3^+^CD56^+^), and T cells. *p < 0.05, Mann–Whitney *U* test.

We postulated that degranulation, as measured by CD107a staining, is a representative marker of cytotoxicity. Compared with healthy livers, liver CD56^bright^, and CD56^dim^ cells from CHCV patients showed an increased cytotoxic capacity (71.1 vs. 42.5%, *p* < 0.05, and 47.0 vs. 18.8%, *p* < 0.05, respectively; Figure 6). Similarly, liver CD3^+^CD56^+^ and T cells from CHCV patients also showed a higher cytotoxic capacity (79.3 vs. 35.0, *p* = 0.03, and 53.5 vs. 26.7%, *p* = 0.03, respectively). In contrast, patients and healthy controls displayed a similar capacity to secrete IFNγ (Figure 6).

### Expression of Differentiation/Activation Markers in NK and T Cells from CHCV Patients

**Figure 7:**
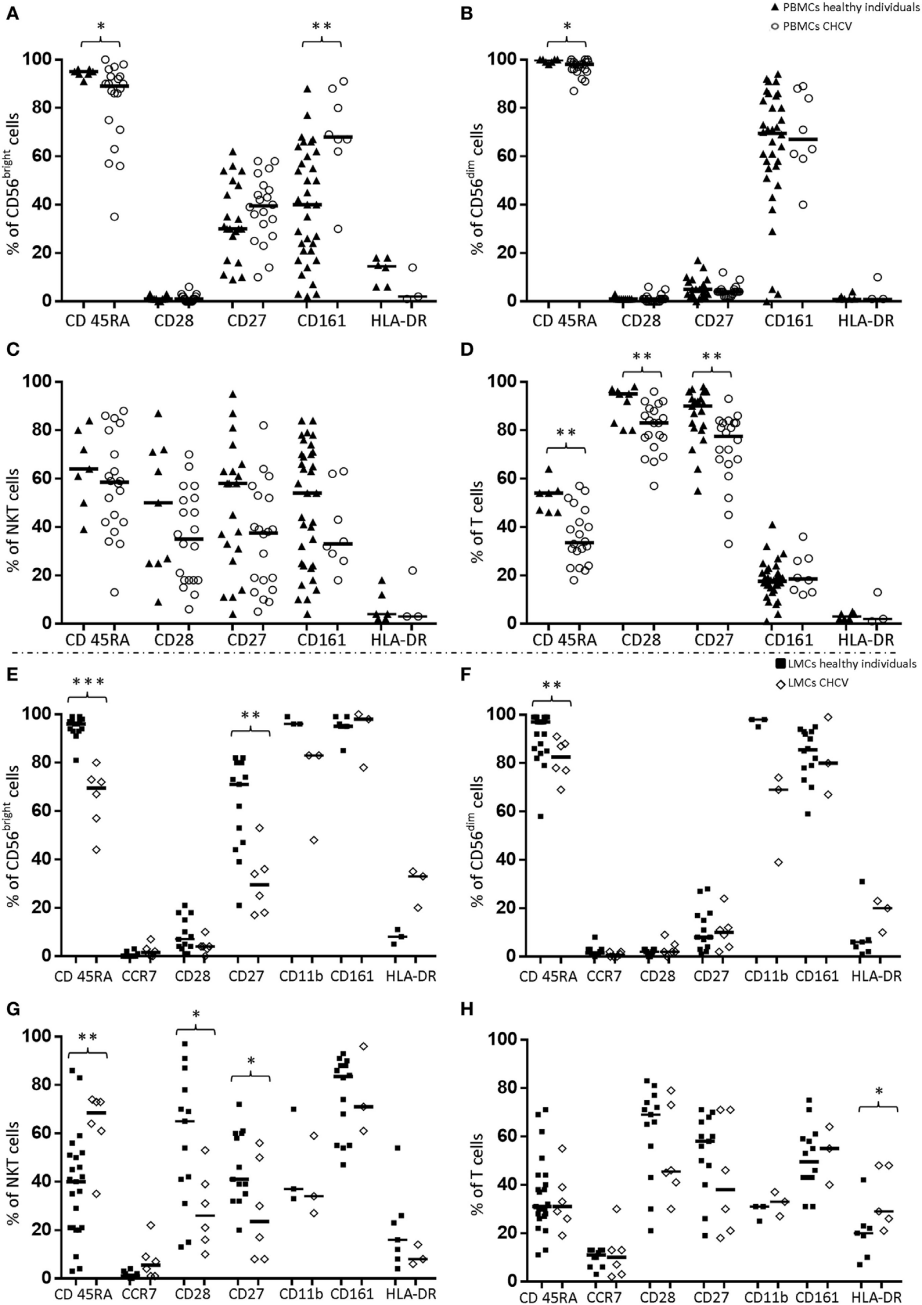
Differences in the expression of differentiation/activation markers in the following cell subpopulations from chronic HCV (CHCV) patients and healthy controls: Peripheral blood mononuclear cell (PBMCs): CD56^bright^ **(A)**, CD56^dim^ **(B)**, NKT (CD3^+^CD56^+^) **(C)**, and T cells **(D)**; liver mononuclear cells (LMCs): CD56^bright^ **(E)**, CD56^dim^ **(F)**, NKT (CD3^+^CD56^+^) **(G)**, and T cells **(H)**. The results are expressed as percentages of positive cells for each killer cell immunoglobulin-like receptor (KIR). *p < 0.05, **p < 0.01, Mann–Whitney *U* test.

Peripheral blood CD56^bright^ and CD56^dim^ cells from CHCV patients showed decreased expression of CD45RA (89.1 vs. 95.4% in healthy controls, *p* = 0.02, and 98.0 vs. 100.0 in healthy controls, *p* = 0.01, respectively; Figures 7A,B). This profile was even more evident in the liver CD56^bright^ and CD56^dim^ cells (69.6 vs. 96.2% in healthy livers, *p* < 0.0001, and 82.5 vs. 97.0% in healthy livers, *p* = 0.009, respectively; Figures 7E,F). Loss of this marker suggests that the cell population included more activated NK cells. On the other hand, CD161 expression was markedly increased in peripheral blood CD56^bright^ cells from CHCV patients (68.0 vs. 40.3% in healthy controls, *p* = 0.0008). Peripheral blood T lymphocytes from patients with CHCV infections displayed decreased expression of the markers CD45RA, CD27, and CD28 (33.8 vs. 54.2% in healthy controls, *p* = 0.004, 83.0 vs. 95.2% in healthy controls, *p* = 0.009, and 77.6 vs. 90.1% in healthy controls, *p* = 0.002, respectively; Figure 7D). A similar trend was observed for the expression of CD45RA, CD28, and CD27 in CD3+CD56^+^ PBMCs (Figure 7C) and in hepatic T and CD3^+^CD56^+^ lymphocytes (45.7 vs. 69.0% in healthy livers, *p* = NS, 38.3 vs. 58.3% in healthy livers, *p* = NS, and 26.0 vs. 65.1%, *p* = 0.03, 23.7 vs. 41.1%, *p* < 0.05, respectively; Figures 7G,H). Thus, the increased frequency of T cells in the livers of CHCV patients is associated with a more differentiated stage.

## Discussion

The role of NK cells in acute HCV infections has been widely evaluated. Most of these studies analyzed either the genomic expression of KIR receptors and their ligands or the expression of NK cell markers in peripheral blood (4, 6, 31, 32). In the present study, we examined the role of NK and T cells in CHCV patients and analyzed the KIR genotype and their protein levels on the surface of peripheral blood and liver cells. As previously reported (16), healthy livers show an enrichment of CD56^bright^ and CD56^dim^ NK cells, which represent approximately half of the LMCs. Compared with healthy controls, PBMCs from CHCV patients featured decreased frequencies of CD56^dim^ NK cells. Similarly, decreased frequencies of CD56^dim^ and CD56^bright^ NK cells were detected in the livers of CHCV patients, but the CD56^dim^/CD56^bright^ ratio was maintained in NK cells. Because the livers of CHCV patients showed significant infiltration of T lymphocytes, we postulated that in contrast with PBMCs, the decreased frequency of NK cells was related to a higher frequency of T cells. In this context, T cells in the peripheral blood and liver from CHCV patients showed a more differentiated phenotype, with fewer naive cells.

As described in previous studies by our group and others (20, 33, 34), key differences are detected between liver-resident NK cells and NK cells from blood, regardless of the liver pathology. This finding was corroborated in the present study because we did not observe phenotypic differences between LMCs from patients with CHCV infections and LMCs from healthy controls. Notably, PBMCs from patients with CHCV infections, and healthy LMCs were obtained from untreated individuals. LMCs that were obtained from patients with CHCV infections before liver transplantation were collected from treated individuals.

The cytotoxic capacity of the liver during CHCV infection has been controversial. Some reports have described decreased cytotoxic capacity (35, 36) as an increase in the number of the CD56^bright^ CD45RA-negative NK cells with high secretory and cytotoxic capacity (37). Likewise, compared with the liver cells from healthy individuals, we detected increased cytotoxic capacity of liver CD56^bright^ and CD56^dim^ NK cells in association with an increase in NKG2C expression and a decrease in CD45RA expression in both NK subsets in patients with CHCV infections. In addition, increased expression of the activating receptor NKG2C was observed, which would reduce the activation threshold of these cells in the presence of their ligand HLA-E a molecule present in Kupffer cells, hepatocytes, and all mononuclear cells (38). The expansion of the CD94/NKG2C activator heterodimer detected in this cohort of CHCV patients is also typically detected in peripheral blood CD56^dim^ NK cells in response to human cytomegalovirus (CMV) infection (39). However, the number of subjects with these expansions did not differ between patients with CMV-seropositive viral hepatitis and corresponding healthy controls (40). Thus, this NKG2C expansion may reflect an underlying CMV infection, because approximately 80% of the Argentinian population is seropositive for CMV.

We detected increased expression of CD107a, a marker of degranulation that is usually used as a marker of cytotoxicity in CD56^bright^ NK cells, CD56^dim^ NK cells, CD3^+^CD56^+^, and T cells from patients with CHCV infections. However, we should be cautious in interpreting these findings because recent studies (41, 42) have reported that liver-resident NK and T cells have the ability to degranulate but lack expression of cytotoxic mediators. Thus, the use of different methods to explore the ability of liver NK cells to kill target cells may explain the discrepancy in the results. In addition, the expression of FcγRIII, the CD16 activating receptor that is an essential mediator of ADCC, is downregulated on liver NK cells, which could explain the decrease in liver ADCC. CHCV infection induces NK cell activation, resulting in ADAM-17-dependent CD16 shedding and subsequent impairments in ADCC (43). Altered ADCC may contribute to the failure to eradicate the HCV infection. In contrast, patients and healthy controls displayed a similar capacity to secrete IFNγ.

The typing of the KIR and HLA genes showed no differences between CHCV patients and controls, and this finding nearly coincided with a previous report from our laboratory (11). However, we detected an increased frequency of the functional alleles of KIR2DS4-FL. We next explored putative associations of the expression of KIR genes and lectin-type C receptors with clinical factors that predict progression to fibrosis and cirrhosis. Interestingly, homozygosity for KIR2DS4-FL, the frequency of which was increased in patients with CHCV infections, was associated with the presence of cirrhosis. This gene dosage effect may have a significant biological impact on patients with CHCV infections (44, 45), because individuals with two copies of the respective gene would exhibit increased expression. Furthermore, the expression of the KIR2DS4-FL gene was increased in CHCV patients with intermediate and high viral loads. In addition, an increased frequency of KIR2DS4 expression was detected in CD56^dim^ NK cells from CHCV patients with elevated transaminase activity levels. Based on these results, we speculate that the increased frequency of the KIR2DS4 genotype may be a consequence of reduced capacity to spontaneously resolve the HCV infection in the acute phase, causing its enrichment in CHCV patients alternatively, KIR2DS4 may be involved in the chronic evolution of the infection by inducing damage to the hepatic parenchyma.

In a previous genetic analysis (11), KIR2DS3 expression was increased in CHCV patients with elevated transaminase levels. We have now determined that KIR2DS3 expression was also correlated with high or intermediate viral loads in patients. Notably, KIR2DS3 was not expressed in any patients with a low viral load. In contrast to our data, KIR2DS3 expression was associated with a low viral load in the absence of KIR2DS5 expression in another study (12). Importantly, KIR2DS3 was present in only 29% of our population (30) but is expressed in 37% of CHCV who progress toward cirrhosis (11). Consistent with our results, KIR2DS3 has been reported to be a risk factor for the development of CHCV infection (46) and has also been associated with the failure to respond to HCV treatment (47). In addition to the lack of an identified ligand, KIR2DS3 is not detected at the NK cell surface, and a KIR gene in linkage disequilibrium with KIR2DS3 may be associated with its clinical effects (48, 49). We also detected decreased expression of KIR3DL1 and increased expression of KIR3DL2 in CD56^dim^ NK cells within the whole cohort of CHCV patients. Of clinical relevance, decreased expression of KIR3DL1 in the presence of its ligand was observed in peripheral blood CD56^dim^ NK cells from patients who did not progress to cirrhosis in the group of early-infected CHCV patients, suggesting that lower expression of KIR3DL1 may delay progression to cirrhosis. Similarly, in the group of late-infected CHCV patients, we observed an association between cirrhosis and lower expression of the strong inhibitory receptor NKG2A in peripheral blood CD56^bright^ NK cells. Consistent with our results, a decreased frequency of KIR3DL1 expression along with a concomitant increase in the surface expression of NKG2D on NK cells was also reported in CHCV patients (36). We also detected that an activated state was related to high necroinflammatory activity and more rapid progression toward fibrosis. These findings suggest that an increased frequency of an activator receptor or a decreased frequency of its inhibitory counterpart is associated with the clinical evolution of the HCV infection (Table 7). According to a recent report (50), genetic factors largely determine the expression of inhibitory receptors, whereas activating receptors are substantially influenced by the environment. CHCV infection is known to induce NK cell activation (43). Therefore, the activation state of liver NK cells explains the restricted expression of KIR-activating genes in liver T cells, as well as the association of KIR-activating genes with CHCV infection.

**Table 7:** Relationship of natural killer (NK) receptors with the clinical evolution of chronic HCV patients.

Factor	NK receptor	Gene/expression
Transaminase activities	KIR2DS4	CD56^dim^
Body mass index	KIR3DL2	CD56^bright^, CD56^dim^, NKT
Evolution to cirrhosis	KIR3DL1^+^ ligand and NKG2A	CD56^dim^, CD56^bright^
Inflammation	CD94/NKG2C	CD56^dim^
Viral load	KIR2DS4-FL and KIR2DS3	Gen
Cirrhosis	KIR2DS4-FL	Gen

The expression of KIR3DL2 was decreased in CD56^dim^ and CD56^bright^ NK cells. These alterations were associated with a high BMI and may be factors associated with the greater progression to fibrosis among CHCV patients.

Our results indicate that an increased frequency of activating receptor expression or a decreased frequency of expression of its inhibitory counterpart may be associated with a worse clinical evolution during the chronic phase of the HCV infection, in contrast to the acute phase of an HCV infection. These changes should be evaluated in the context of a more differentiated/activated state of peripheral blood and liver T cells and NK cells, and their increased capacity to degranulate may reflect an increase in their cytotoxic activity. A proposed hypothesis is that in patients with CHCV infections, individuals who are more likely to have activated NK and/or CD3^+^ CD56^+^ cells would exhibit evolution of the disease. Further studies are required to confirm our results concerning the role of the expression of KIR genes in the progression of HCV infection.

## Ethics Statement

This study was carried out in accordance with the recommendations of the ethical guidelines of the 1975 Declaration of Helsinki with written informed consent from all subjects. All subjects gave written informed consent in accordance with the Declaration of Helsinki. The protocol was approved by the Investigation and Ethics Committee and Institutional Review Board of the Hospital de Clínicas José de San Martín. All samples obtained during the liver transplant were identified by a transplant procedure number provided by INCUCAI without the name of the donor. None of the transplant donors were from a vulnerable population and all donors or next of kin provided written informed consent that was freely given.

## Author Contributions

AP and LF have made substantial contributions to the conception and design and the acquisition, analysis and interpretation of the data. LF also obtained funding. MD, AM, SB, and SM helped with the analysis and interpretation of data. MD, SM, SP, HF, and LP help providing samples and study supervision.

## Conflict of Interest Statement

The authors declare that the research was conducted in the absence of any commercial or financial relationships that could be construed as a potential conflict of interest.

## Supplementary Material

Figure S1 Illustrative scheme for the analysis of killer cell immunoglobulin-like receptor (KIR) expression in peripheral blood mononuclear cells **(A)** and liver mononuclear cells (LMCs) **(B)** on CD56^dim^ natural killer cells in a healthy individual. The expression of 2DS3 was deduced after subtracting the expression detected by anti-2DL1/2DS1/2DS3 (1) the frequency detected by 2DL1/2DS1 (2) 0.2DS1 was deduced by subtracting the expression of 2DL1 (3) from the expression detected using the 2DL1/2DS1 antibody (2). The expression of 2DL2/2DS2 was inferred after subtracting the expression detected by the anti-2DL3 antibody (5) to the expression detected by the anti-2DL2/2DS2/2DL3 antibody (4). In all cases, the presence of the KIR gene was verified.

## Abbreviations

CHCV, chronic HCV; LMCs, human liver mononuclear cells; NK cell, natural killer cell; KIR, killer cell immunoglobulin-like receptor; NKT cell, natural killer T cell.
