# Supplementary figures and images for: The Clinical Features of Patients with Chronic Hepatitis C Virus Infections Are Associated with Killer Cell Immunoglobulin-Like Receptor Genes and Their Expression on the Surface of Natural Killer Cells
Source: Front Immunol. 2018 Jan 5;8:1912. doi: 10.3389/fimmu.2017.01912 (PMC5760500; doi:10.3389/fimmu.2017.01912)

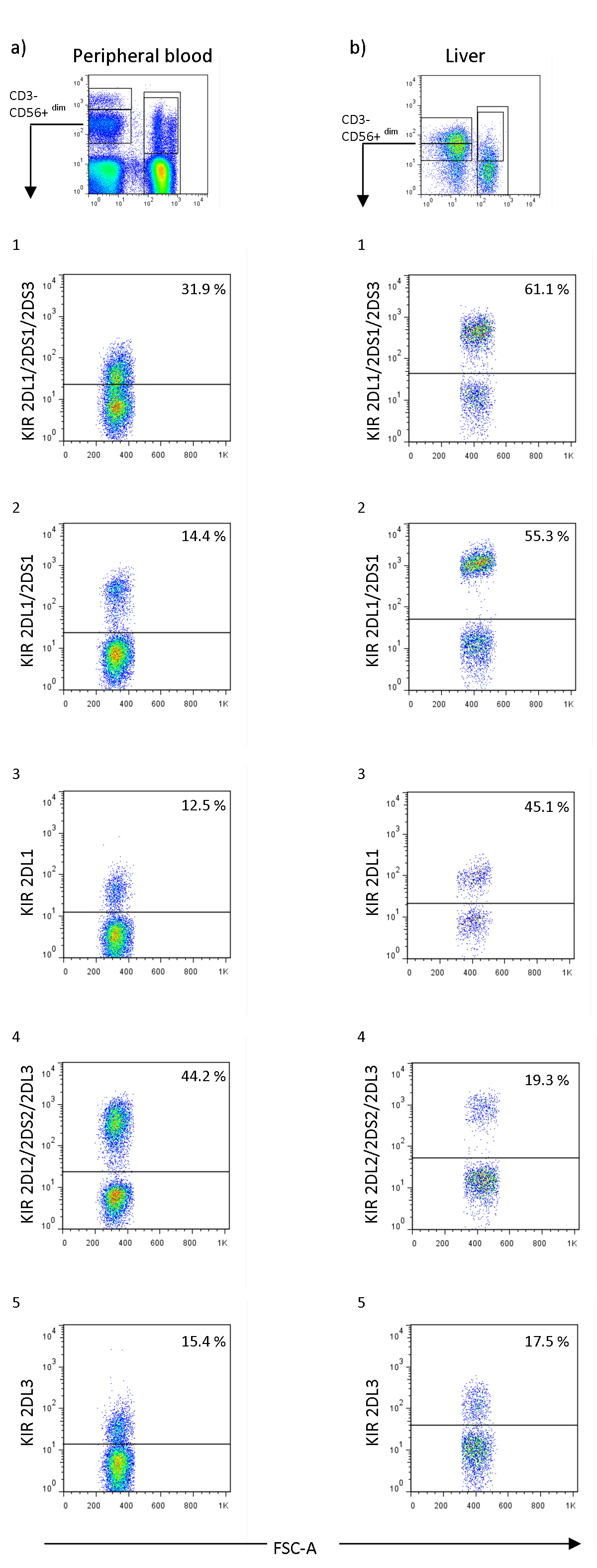

Supplement: Figure S1 — Illustrative scheme for the analysis of killer cell immunoglobulin-like receptor (KIR) expression in peripheral blood mononuclear cells (A) and liver mononuclear cells (LMCs) (B) on CD56dim natural killer cells in a healthy individual. The expression of 2DS3 was deduced after subtracting the expression detected by anti-2DL1/2DS1/2DS3 (1) the frequency detected by 2DL1/2DS1 (2) 0.2DS1 was deduced by subtracting the expression of 2DL1 (3) from the expression detected using the 2DL1/2DS1 antibody (2). The expression of 2DL2/2DS2 was inferred after subtracting the expression detected by the anti-2DL3 antibody (5) to the expression detected by the anti-2DL2/2DS2/2DL3 antibody (4). In all cases, the presence of the KIR gene was verified. [file image_1.tif]
